# Internet-Based HIV and Sexually Transmitted Infection Testing in British Columbia, Canada: Opinions and Expectations of Prospective Clients

**DOI:** 10.2196/jmir.1948

**Published:** 2012-03-06

**Authors:** Travis Salway Hottes, Janine Farrell, Mark Bondyra, Devon Haag, Jean Shoveller, Mark Gilbert

**Affiliations:** ^1^BC Centre for Disease ControlVancouver, BCCanada; ^2^Faculty of Health SciencesSimon Fraser UniversityBurnaby, BCCanada; ^3^School of Population and Public HealthUniversity of British ColumbiaVancouver, BCCanada

**Keywords:** HIV, human immunodeficiency virus, sexually transmitted diseases

## Abstract

**Background:**

The feasibility and acceptability of Internet-based sexually transmitted infection (STI) testing have been demonstrated; however, few programs have included testing for human immunodeficiency virus (HIV). In British Columbia, Canada, a new initiative will offer online access to chlamydia, gonorrhea, syphilis, and HIV testing, integrated with existing clinic-based services. We presented the model to gay men and other men who have sex with men (MSM) and existing clinic clients through a series of focus groups.

**Objective:**

To identify perceived benefits, concerns, and expectations of a new model for Internet-based STI and HIV testing among potential end users.

**Methods:**

Participants were recruited through email invitations, online classifieds, and flyers in STI clinics. A structured interview guide was used. Focus groups were audio recorded, and an observer took detailed field notes. Analysts then listened to audio recordings to validate field notes. Data were coded and analyzed using a scissor-and-sort technique.

**Results:**

In total, 39 people participated in six focus groups. Most were MSM, and all were active Internet users and experienced with STI/HIV testing. Perceived benefits of Internet-based STI testing included anonymity, convenience, and client-centered control. Salient concerns were reluctance to provide personal information online, distrust of security of data provided online, and the need for comprehensive pretest information and support for those receiving positive results, particularly for HIV. Suggestions emerged for mitigation of these concerns: provide up-front and detailed information about the model, ask only the minimal information required for testing, give positive results only by phone or in person, and ensure that those testing positive are referred for counseling and support. End users expected Internet testing to offer continuous online service delivery, from booking appointments, to transmitting information to the laboratory, to getting prescriptions. Most participants said they would use the service or recommend it to others. Those who indicated they would be unlikely to use it generally either lived near an STI clinic or routinely saw a family doctor with whom they were comfortable testing. Participants expected that the service would provide the greatest benefit to individuals who do not already have access to sensitive sexual health services, are reluctant to test due to stigma, or want to take immediate action (eg, because of a recent potential STI/HIV exposure).

**Conclusions:**

Internet-based STI/HIV testing has the potential to reduce barriers to testing, as a complement to existing clinic-based services. Trust in the new online service, however, is a prerequisite to client uptake and may be engendered by transparency of information about the model, and by accounting for concerns related to confidentiality, data usage, and provision of positive (especially HIV) results. Ongoing evaluation of this new model will be essential to its success and to the confidence of its users.

## Introduction

Several jurisdictions have implemented Internet-based sexually transmitted infection (STI) testing programs, with good uptake and reach into untested populations [1-11]. This approach is consistent with broader efforts to complement existing face-to-face sexual health services with online interventions [12]. Various models for publicly funded Internet-based STI testing have been explored. Most initially engage clients through a website. Specimen collection may then be facilitated either by mail, in which case samples are self-collected at home [5,11], or by providing a requisition that can be presented at designated specimen collection sites or laboratories [7,8]. Some programs continue to deliver results by telephone or face to face [1,4]; however, the feasibility and acceptability of online result delivery, particularly for chlamydia and gonorrhea, has been demonstrated [6,10,13]. The majority of online testing services implemented to date are broad, population screening interventions for chlamydia, predominantly targeting youth [4,10,11,14-16]. Notably, few programs have incorporated testing for human immunodeficiency virus (HIV), and few have targeted gay men and other men who have sex with men (MSM) [7,8].

 In British Columbia, Canada, a new program is under development at the BC Centre for Disease Control (BCCDC) to provide online access to chlamydia, gonorrhea, syphilis, and HIV testing, through a model integrated with existing clinic-based services. This program will initially be offered in a pilot phase to clients attending two urban STI clinics—where approximately 10,000 clients are screened annually—and additionally to MSM in Vancouver. After evaluating the results of the pilot phase, this service is intended to expand to other parts of the province, with further targeted promotion to groups that experience high rates of STI and HIV. The goals are to increase test uptake and frequency and to ease demand on clinic-based testing services. The current model invites prospective users to visit a secure website where they will create an account, review pretest information, answer a series of questions (related to, for example, symptoms, recent exposure to STI/HIV, recent sexual behaviors, and history of STI diagnosis), and then print a laboratory requisition. Clients will present to designated specimen collection sites to give blood and urine samples; in the Vancouver area, these sites offer greater flexibility in hours and locations than existing STI clinics. Those who test positive will be contacted by a nurse, who will deliver results by phone or in person, consistent with current clinical practice at the BCCDC. Negative results will be viewable online via the same secure website. Prior to using the service, clients will review an overview of the testing process, including methods for results delivery, on the website. The service will be free of cost for all clients, though cost recovery mechanisms (eg, through the provincial public medical service billing system) will be explored as the service expands.

 The importance of formative evaluation to provide end-user input at early stages in the development of novel eHealth interventions is well established [17-19]. Research related to the development of Internet-based preventive care management systems in sexual health as well as other domains (eg, diabetes, arthritis) has illustrated the need first to identify the right group of users for a new online intervention, then to ensure that features of the new service are well tailored to the intended user group and mindful of their most significant concerns—for example, confidentiality in the case of sexual health [5,20,21]. While the acceptability of some Internet-based STI testing models has been demonstrated elsewhere [5,6], we anticipated concerns that may be unique to the British Columbia setting (eg, within the context of a publicly funded health care system) and model, which includes multiple infections—notably HIV—and is integrated with clinic-based services. Anonymous HIV testing is not available in British Columbia, though to afford additional protection of privacy, clients seeking HIV testing have the option to suppress their name and address when a positive result is reported to public health; some STI clinics in the province furthermore allow clients to test using pseudonyms [22]. With respect to delivery of results, clients of the Provincial STI Clinic at BCCDC are contacted by a nurse if any result is positive but may otherwise telephone the clinic to receive their test results. Awareness and utilization of these existing testing options may influence expectations of the online testing service.

Recent in-depth interviews with youth 15–24 years of age in British Columbia have suggested that this population appreciates online sexual health services for the convenience and privacy they afford; however, youth had low tolerance for technologies perceived to be outdated, such as the requirement to print a laboratory requisition form [23]. The current study expands on these findings through formative research with adult MSM and STI clinic clients in Vancouver. We conducted a series of focus groups to gauge initial reactions to the British Columbia Internet-based STI/HIV testing model, identify components of the model that require modification, and describe end users’ overall perception of and intention to use the service. Based on other studies and formative work with community-based organizations in British Columbia, we sought to understand particular concerns related to confidentiality and provision of results, as well as ways to create and maintain trust in the service.

## Methods

### Participants

Focus group attendees were MSM and clients already accessing in-clinic STI testing services (ie, members of target populations to be included in the pilot phase of the British Columbia Internet testing service). MSM were recruited into one of three focus groups through online classifieds and gay news site advertising (6/20, 30%), community agency email lists (5/20, 25%), posters at gay community organizations and businesses (4/20, 20%), and word of mouth (5/20, 25%). Clinic clients were recruited into a further three focus groups through in-clinic flyers (2/19, 11%), emails to clients who had previously consented to be contacted for research (14/19, 74%), and word of mouth (3/19, 16%). All participants were 19 years of age or older, resided in the greater Vancouver area, and gave written informed consent. This study was approved by the Research Ethics Board of the University of British Columbia.

### Procedures

**Figure 1:**
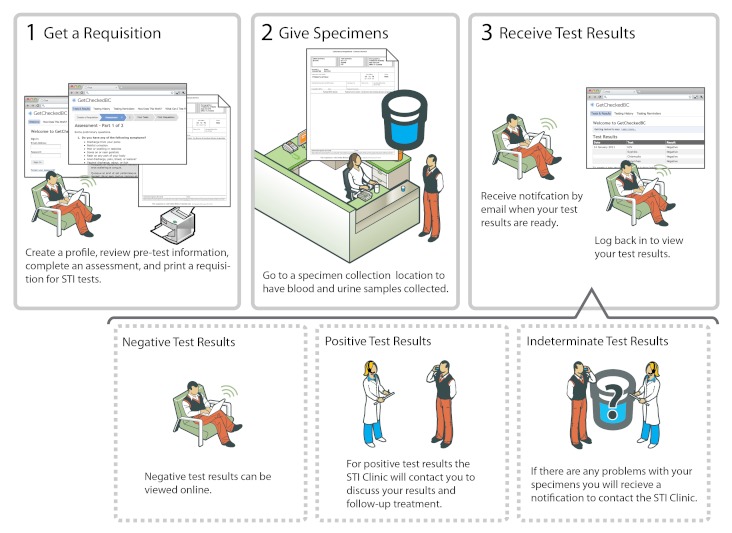
Proposed British Columbia Internet-based sexually transmitted infection (STI)/human immunodeficiency virus testing model, as presented to focus group participants.

Focus groups were audio recorded and lasted 1.5 to 2 hours; they were conducted at the BCCDC in private meeting rooms with one moderator and one observer who took detailed field notes. Upon arriving participants completed a brief, anonymous questionnaire. Prior to discussion participants were reminded to respect the confidentiality of their peers, and participants were never asked to share their name in front of the group. At the start of each focus group, participants discussed their Internet use and past experiences with accessing STI and HIV testing. They were then given a brief (<5 minutes) description of the model (Figure 1), after which the moderator answered clarifying questions. A structured interview guide was used to address the following domains throughout the remaining discussion: willingness to provide personal information online, ways to engender trust in the service, comfort with different ways of delivering results, interest in specific features, appeal of the service, and willingness to use the service. Participants received a $25 cash honorarium.

### Analysis

An analyst first reviewed the audio recordings and field notes from each focus group to ensure that every statement was captured; this analyst was distinct from the observer who took notes for that particular group. A consensus-based coding scheme was initially developed based on a literature review and subsequently modified to reflect new concepts that emerged throughout the analysis period. Two team members independently reviewed field notes to apply codes from the scheme. They then jointly used a scissor-and-sort technique to analyze the data: printed and coded field notes were cut so that individual statements could be sorted and re-sorted to explore common themes [24]. Analysis was iterative, covering all codes from the scheme and ultimately giving analysts a sense of frequency or extensiveness of each theme. Analysts returned to audio recordings to transcribe quotations of illustrative comments. Except where noted, results reported here include themes identified across two or more focus groups. Results also include descriptions of connections across concepts based on salient themes that we identified as the analysis progressed.

## Results

### Participant Characteristics

A total of 39 people participated in six focus groups (4–9 participants each) between February 25 and May 5, 2011. Most were men (32, 82%) who identified as gay (19, 49% of total sample), bisexual (4, 10%), or two-spirit (2, 5%). A total of 28 (72%) reported having postsecondary education. All participants had experience with HIV or STI testing, and nearly all were active Internet users: most reported being online 15–40 hours per week, and some described themselves as “[an] Internet addict” and “permanently wired in.” None of the participants were familiar with the British Columbia Internet-based STI/HIV testing initiative prior to attending, and few had heard of Internet-based testing services offered elsewhere. Table 1 describes additional participant characteristics.

**Table 1:** Sociodemographic characteristics, testing behaviors, and access to relevant technology among focus group participants (N = 39).^a^

Variable		n	%
**Age range (years)**			
	20–29	10	26%
	30–39	5	13%
	40–49	14	36%
	≥50	10	26%
**Gender**			
	Male	32	82%
	Two-spirit	3	8%
	Female	4	10%
**Country of birth**			
	Canada	34	87%
**Sexual orientation**			
	Gay	19	49%
	Bisexual	4	10%
	Two-spirit	2	5%
	Straight	12	31%
	Unknown	2	5%
**Highest level of education completed**			
	Elementary school	1	3%
	High school	10	26%
	University	23	59%
	Graduate school	5	13%
**Testing history**			
	Ever tested for HIV^b^	38	97%
	Ever tested for STI^c^ (other than HIV)	36	92%
	Tested for HIV or STI in past year	29	74%
**Access to technology**			
	Private Internet-connected computer	39	100%
	Printer that can be used to print personal information	31	79%

^a^ Based on self-report through anonymous questionnaire.

^b^ Human immunodeficiency virus.

^c^ Sexually transmitted infection.

### Current Barriers to In-Clinic Testing

Participants articulated numerous barriers to in-clinic STI/HIV testing (Table 2). The most common were embarrassment and stigma. Embarrassment was often described as discomfort with talking to a clinician about sex, sexuality, or STI and HIV tests. Stigma was a broader, more complex issue that permeated most topics of discussion and was raised in every focus group. Stigma was described in terms of perceived judgment, not only from the provider but also from those who might see them or know they were going to get tested. While most participants had recently accessed testing themselves, they recounted past experience or knowledge of friends’ experiences in expressing how broader social stigma leads to avoidance of testing. As one middle-aged man explained, “I’ve been in situations with the clinic before where you get this judgment and this fear factor built up and you just don’t want to talk to them anymore and you end up just not going back.” Others discussed frustration with in-person testing, citing long wait times—both to get an appointment and to be seen after arriving at the clinic—and difficulty returning to the clinic for results. Finally, limited access to competent or sensitive sexual health services was another barrier discussed. This may include not having a family doctor or dissatisfaction with a family doctor; for example, some expressed concern that their doctor may not know the right tests to offer. One participant told of a family doctor who was reluctant, even uncomfortable, to offer an HIV test: “I went to my family doctor and told him I wanted an HIV test and he was like ‘why?, you’re not gay,’ and um obviously, well, yah I am and I do...I had to go to a hospital to go get tested.” Participants were also mindful of those who live in rural areas or far from the city center, who may not have access to nonjudgmental or gay/lesbian/bisexual/transgender-friendly STI clinics as an alternative to going to see a family doctor.

### Perceived Benefits of Internet Testing

In this context, Internet-based testing was thought to offer the potential to circumvent some of these existing barriers through anonymity, access, convenience, and control (Table 2). Participants repeatedly described anonymity, or the “faceless experience,” as perhaps the greatest promise of the Internet in relation to sexual health services. By ordering tests online, participants felt that they would avert some of the aforementioned worries about someone seeing them walk into an STI clinic:

This is definitely a service I would use, not only for the convenience factor but I mean, no matter how old we are, it’s still an embarrassing issue for a lot of people. Like he was saying, there’s the STI clinic, and so what I do is look around and see what traffic is on the road...because it’s embarrassing for me.

The facelessness of the Internet may further facilitate clients’ comfort with providing personal information. As one young woman observed:

I’d be definitely more comfortable doing it [answering questions about sexual behaviors] online because I think people have a tendency to underestimate when in person, because I think as you said the fear, or it’s embarrassing when you’re asked how many sexual partners you’ve had, people will tend to go on the low end, you know, it’s just human nature, so I’d rather do it online probably.

The ability to access Internet testing any time of the day or night was also emphasized as a key benefit. In light of the problems some individuals had with getting to the clinic during business hours to retrieve results, online access to test results offers a striking advantage. One clinic client explained that “being able to get a negative test result by logging in and saying oh there it is, really provides a lot of convenience...the easier you make it to get a negative test result, the more people will be tempted to use the service.” A 20-something-year-old gay man highlighted the client-centered control of Internet-based services as a major appeal:

I can’t speak for all people, but certainly for a lot of people in my generation, well our generation, certainly, it’s just um easier. You get to, it’s the email thing, your results are ready, you check it, and then you can call someone...I prefer it because...it sort of gives me agency over my own health care in a way, like, in the sense that here’s the information, I can do with it what I choose, rather than relying on some doctor or some nurse that I may not know to sort of decide how they’re going to do it and sort of be in that emotional space, you know. I can then decide how I approach it.

Using the Internet to offer testing was furthermore thought to be beneficial for *both* the client and the provider, in that it will standardize the service clients receive—taking away some of the variability encountered when seeking in-clinic testing—while allowing providers to focus their time on delivering results, treatment, and follow-up, with the potential to also reduce costs.

**Table 2:** Barriers to in-clinic sexually transmitted infection and human immunodeficiency virus testing, and corresponding benefits of Internet-based testing, as expressed by participants.

Existing barriers to testing	Perceived benefits of Internet-based testing
Embarrassment of talking to a clinician about sexual health concerns; and stigma or perceived judgment associated with seeking testing	Anonymity or faceless experience of ordering tests online
Long wait times at the clinic	Immediate access to website
Difficulty getting to sexually transmitted infection clinic during business hours	24-hour availability of Internet; extended/flexible hours of specimen collection (laboratory) sites
Dissatisfaction with/lack of family doctor	Standardized service, controlled by the client

### Building Trust

When participants were presented with the Internet testing model (Figure 1), many initially expressed some hesitation or confusion (Table 3). While some of the previously mentioned anonymity-related concerns may be mitigated by an online platform, new misgivings about the provision of personal information were heightened with the use of the Internet:

I think dealing with people’s fears around this is a critical component of it. And anonymity is going to be kind of a position people will start from, and then they’ll sort of move to a point of being more comfortable.

For some this reluctance to provide personal information was traced to fears about security; one participant explained that “I would say, first of all, you know, I wouldn’t be comfortable putting any personal information into a website like this where I know someone could hack into it or something.” Others wanted to know what the service provider would be doing with their information, where it would be stored, and why it was being collected. In response, however, participants offered suggestions to allay these concerns about sharing personal information, as illustrated by the following quotes and further outlined in Table 3.

If you’re going to use an email address to register, you may want to say something like go to Gmail or Hotmail and create a new, anonymous email account that you only use for this, if you want to have more privacy, if you don’t feel comfortable using your own personal email account.

For people who obviously want to do this confidentially...I’m assuming people are going to be using it because they don’t want people to see them going to a clinic...when you refresh the page, and someone comes by and someone is being snoopy, ‘why were you on that website?,’ you know kind of thing, I don’t know if there’s computer technology for back browsing or to clear the cache so when you leave that site there’s no way to tell them you were on that site.

A few participants expressed concern that someone might maliciously enter another person’s name or email address when creating an account. Participants consequently suggested that email addresses be verified by sending a confirmation email and requiring a response before activating the account. Most participants were ultimately willing to give information required for STI/HIV testing but not more than is absolutely necessary. They also expected to be told why they were being asked for each piece of information, repeatedly suggesting that a description of the use and purpose of all data collected be clearly described for prospective clients up front, in advance of registering for the service.

 Other concerns with the model related to lived or perceived experiences with positive test results, particularly for HIV; these concerns were more prevalent in the focus groups with gay men and other MSM. A few participants worried that testing online would mean that clients would not receive all of the pretest information they thought they needed:

How do you know somebody has actually read all the information? I click agree to the conditions, right, like when you order software or whatever. Nobody reads all those things, but you have to click it to get through.

There was also frequent concern for what it would be like to get a positive result using the Internet testing service, and participants were eager to suggest extra measures to protect those receiving positive results. One person suggested that all clients be required to receive all four tests (ie, chlamydia, gonorrhea, syphilis, and HIV) so that if they are notified that a result is positive they wouldn’t know for certain that it was their HIV result. This suggestion was liked by some participants as a way to reduce anxiety for testers but disliked by others because it was regarded as contrary to the otherwise client-centered nature of the model. To remedy this, many participants suggested providing positive HIV results in person or by phone only, and providing Web links to other care and support, including community-based peer services and counseling:

I think one of the things that could be offered through Internet resources is uh immediate access for people who are getting devastating results. Where do you go next? What do you do? In the first 48 hours you need critical care for these people.

Participants also talked about the anxiety experienced while waiting for a test result and worried that such anxiety could be exacerbated when testing online; thus, many participants urged that resources and referrals be provided at the time of testing (via the website or laboratory requisition form), as well as at the time of diagnosis.

 When asked how trust in the service might be gained, participants noted the importance of professionalism and of adhering to standardized guidelines. As outlined above, transparency of information and practices is key. Three participants, across multiple focus groups, inquired about whether evaluation or research would be done to ensure the service is meeting its goals and is acceptable to users. Additionally, the legitimacy of the organization sponsoring the program was noted to be relevant in gaining trust in the new service (eg, BCCDC as a government organization).

**Table 3:** Concerns with Internet-based sexually transmitted infection/human immunodeficiency virus (HIV) testing and corresponding strategies for mitigation expressed by participants.

Concern	Suggestions for mitigation
Reluctance to provide personal information online	Ask only for information required for testing Explain rationale for other data collected Validate email address when creating a new account
Distrust of security of data provided online	Describe security measures of website up front Explain additional measures client can take (eg, private browsing, clear cache/history)
Ensuring comprehensive pretest counseling	Remind clients of the option of coming to a clinic for face-to-face pretest discussions Include detailed pretest information on the website
Support for those waiting for results and receiving positive results, particularly for HIV	Do not provide any positive results online Provide links to referrals, including counseling and support services in community, at time of testing (via website)

### Expectations of the Service

In general, most participants expected that the Internet testing service would offer features similar to other commonly used Web-based services and in particular would provide continuous online service delivery. This included a strong interest in booking in-clinic appointments and getting prescriptions for STI treatments online. When presented with the model, several people noted the requirement to print a laboratory form and suggested that the requisition data instead be sent electronically, either to the client’s smart phone or directly to the laboratory itself:

What if you were provided with a verification code, say to my iPhone, to my email address, that I could just take to [the lab], show them the code, and they would have the information in their system?...it could go direct to their forms.

Nearly all participants were interested in receiving result notifications (ie, messages indicating that results are ready) and testing reminders, either by email or by text message, though many emphasized that clients should be able to control when and how such notifications are sent, bearing in mind the potential for breaches in confidentiality, through email especially. Repeatedly participants stated that they would like to have options with regard to nearly every aspect of using the service, and in particular when receiving communications:

[Regarding how would you like to be notified of results] I’d rather it just be like, either or, check online, and maybe click something if you prefer to be notified by phone...but certainly there should be the option as well for people who just want to be notified over the Internet, on their own terms.

Participants also believed that standards of service for online testing should be similar to those in the clinic, and where they differ (eg, for particular STI tests not being offered online), clients expect to be given referrals to other places for testing. Likewise, when asked what questions they would expect to be asked before testing online, participants responded that they would expect to see the same questions they would be asked in a clinic. As one participant explained:

I’d say with regard to the risk assessment though there would be no reason to have it any different than the questions they ask you when you come in here [STI clinic], you know like how many partners, the whole thing they go through.

In response, another participant noted that “when you give answers here [at the BCCDC] they type them into a computer database anyway.”

### Barriers to Use of Online Testing Service

Across focus group discussions, a few noteworthy barriers to use of the Internet testing service were elicited. These limitations predominantly reflected concerns over levels of technical or English-language literacy. Although 79% of participants reported access to a printer where they could print confidential information, many raised the lack of a personal printer and the expense and hassle of obtaining ink and printer paper as barriers to using this service. A middle-aged heterosexual male participant elicited agreement from several others in his focus group when he described a typical situation:

My only printer is at work. [moderator: What would it be like to print a requisition like this at work?] It’s easy, but I mean, mine’s a group printer. There’s eight of us using one printer, and if I’m not up and at that printer, they’re going to look at it and ask, ‘what the hell is this?’

When asked whether this would ultimately dissuade them from using the service, most acknowledged that it would not, though it would likely be perceived by some as an annoyance. Beyond the concerns with printing, others noted varying levels of comfort with computers and with the Internet as potential barriers. Focus group attendees furthermore highlighted limited English-language skills, among both native and nonnative speakers, suggesting that instructions and information on the site be written in a style mindful of those with more basic levels of language literacy and health literacy.

### Uptake

Most participants said they would use the Internet testing service or recommend it to others. Those who indicated they would be unlikely to use it generally lived near an STI clinic and therefore had convenient access to testing, or routinely saw a family doctor with whom they were comfortable testing (eg, HIV-positive individuals who were regularly receiving screening and other care and treatment services through a primary care provider). Some also suggested that Internet testing may not be well suited for people such as first-time testers, who may have heightened anxiety or questions that are best handled face to face.

 Participants expected that the service would provide the greatest benefit to individuals who do not have access to sensitive sexual health services (for example, those living outside the city center or in rural or remote areas), are reluctant to test due to stigma (eg, youth or non-gay-identified MSM), want to start the process of testing immediately (eg, those who had a recent possible exposure to an STI/HIV), or may simply forget or put off going for routine testing. As one participant expressed, “I’m someone who procrastinates a bit, and this would remind me to get going, get tested every...however often*.*”

## Discussion

This study of gay, bisexual, and two-spirit men and STI clinic clients in Vancouver, Canada revealed generally high levels of enthusiasm for Internet-based STI/HIV testing. Focus group attendees started from a point of reluctance toward the Internet testing concept, raising concerns related to confidentiality, data usage, and provision of positive (especially HIV) results, but gradually moved to a point of confidence and comfort after seeing that these concerns were accounted for in the present model. Trust in the service is thus a prerequisite to client uptake and may be engendered through transparency of information about the model, by providing positive test results preferably in person (in the case of HIV) or by phone, and by ensuring that provisions are in place to link those testing positive or waiting for results with referrals for care, including counseling and community-based support services. Notably, for each of the most prevalent concerns elicited, participants themselves identified a mitigation strategy (Table 3). Other suggestions generated from this study—for example, inclusion of online prescriptions—may not be feasible for the pilot phase of the service but are useful in long-term strategic planning for program expansion.

 The proposed Internet-based service was described by potential users through a discussion of tensions and trade-offs. Losing the face-to-face interaction of traditional clinic settings was welcomed when it meant a gain in anonymity but was questioned in terms of the ability to provide support for those receiving positive test results. Generally, clients expected that the online service would take care of them, offering the quality of care equivalent to that provided in a clinic; however, they wanted to see justification for all questions asked of clients via the website and resisted the collection of extraneous personal data. Likewise, some participants expressed a desire for paternalistic features (eg, requiring all clients to test for all four infections), while others demanded a more client-centered model where they could test and receive results in a way of their choosing. These trade-offs suggest that Internet testing for STI and HIV requires careful balancing to respect divergent viewpoints. Ultimately this service cannot accommodate *all* client preferences, though we may heed these requests by providing options where possible. For example, while clients will not be able to choose to receive positive results online, they may be able to opt in or out of email notifications concerning negative results.

 Our findings were generally consistent with those of others who have assessed the acceptability of online STI screening services [5,6,23]. Concerns over confidentiality and data usage are common in relation to sexual health services [25-27] and perhaps not surprising given the stigma and shame associated with STI and HIV, as articulated by our participants themselves. Privacy- and health data-related concerns have been heightened in recent years in British Columbia, in response to an initiative to both centralize and expand access to electronic medical records in the province [28], as well as increasing criminal cases for HIV nondisclosure [29]. As found in qualitative studies of British Columbia youth [23], the adults sampled in our study expressed an expectation that online services be totally online and balked at the requirement to print a form to use the service. Unlike most other Internet testing models that have been described in published research, ours includes the option to test for HIV. Participants in our focus groups praised the increased accessibility to HIV testing afforded by this model but also raised concerns with the experience of testing for HIV in a new setting, particularly one that does not include a face-to-face consultation. A disproportionate amount of the anxiety around provision of results was centered on the HIV test, especially among gay and bisexual men and other MSM. This was a noteworthy concern but not an irreconcilable one, and various strategies for mitigation were elicited, as already noted.

Soliciting input from end users through formative research is recommended at early stages of development of novel, Internet-based health services [17,18], though there are compelling challenges to achieving this in the setting of understaffed and underfunded small governmental projects. Our study demonstrates how such qualitative research can nonetheless provide valuable results through the use of efficient methods such as field notes (in lieu of full data transcription) and scissor-and-sort analysis techniques. Such methods preclude detailed analysis of themes that may be pursued in traditional qualitative research but allow for rapid cycles of recruitment, interview or focus group conduct, analysis, and interpretation, the rehearsal of which is essential to the success of programs like ours. We have thereby been able to incorporate the key findings highlighted above—and in particular the mitigation strategies outlined in Table 3—into the British Columbia Internet testing model in real time.

The participants in this study were largely Internet-savvy, highly educated, urban gay men who were already experienced with testing. Thus, our findings are helpful in tailoring this intervention for the more limited pilot phase but may not be generalizable to other groups—notably those that are not currently testing for STI/HIV, a population that will be of keen interest during the provincial expansion of the program and that may have particular concerns when first testing through an online platform. Participants themselves noted that Internet testing likely holds the greatest benefit for those living outside the city center, who have limited access to sensitive sexual health services. Further focus groups with traditionally underserved and marginalized populations are planned and will aid in the development and scale-up of this service.

 As illustrated by the data presented here, Internet-based STI/HIV testing is a potentially powerful complement to existing clinic-based services. Despite the rapid growth in online testing programs globally, these services remain unfamiliar to most target users in British Columbia. In light of its differences from traditional testing services, fully understanding the needs and expectations of prospective clients is imperative, as is providing them with clear, up-front information about the new model, along with justification for its particular functions and features. Much of the research on Internet testing has explored feasibility and acceptability [30]; more data are now needed to determine its effectiveness in relation to other outcomes such as frequency of testing, uptake among those not already testing, and uptake among those most at risk of infection.

## Abbreviations

BCCDC: British Columbia Centre for Disease Control
HIV: human immunodeficiency virus
MSM: men who have sex with men
STI: sexually transmitted infection
